# A Generalized AI View of Tricopeptide Repeats: What’s in a Name

**DOI:** 10.3390/ijms262311649

**Published:** 2025-12-01

**Authors:** Sailen Barik

**Affiliations:** Independent Researcher, 8220 Walter Court, Mobile, AL 36695, USA; s.barik@csuohio.edu or barikfamily@gmail.com; Tel.: +1-251-454-1255

**Keywords:** tricopeptide repeat, helical domain, superhelix, artificial intelligence, alphafold, protein motif, protein–protein interaction, RNA-protein interaction

## Abstract

Tricopeptide repeats refer to 30 or more amino acid (aa) repeats, of which the best studied ones are 34-aa and 35-aa long, named Tetratricopeptide and Pentatricopeptide repeats, respectively, and abbreviated as TPR and PPR. Recently, 37-aa and 38-aa repeats (Heptatricopeptide, HPR; Octatricopeptide, OPR) have been reported, but 36-aa repeats or repeats outside the 34–38 range (such as 33-aa or 39-aa) are apparently missing. This review is an analytical discourse of the structural and functional commonalities as well as differences among all tricopeptide repeats. In structure, the use of Artificial Intelligence (AI)-based prediction and experimental 3D structures revealed that regardless of the number of amino acids, these repeats are all alpha-helical in nature, whereby the tandem helices are joined by relatively flexible linkers or spacers to form a superhelix. In function, many tricopeptide repeats bind specific RNA, thus playing important roles in RNA processing and stability. The specificity is determined by the interaction between specific amino acid residues with the nucleotides in the RNA, while the helices offer a scaffold that holds the interacting residues in position. Detailed analysis of various known TPR and PPR revealed conserved amino acids at specific positions, such that they serve as signature motifs. Moreover, extra helices upstream or downstream of the repeat domains often maintain the continuum of the superhelical vortex. Evidently, the overall helicity and the presence of critical amino acid residues in strategic places are more important for the biological function of the tricopeptide repeats than the exact amino acid length of the repeat.

## 1. Introduction

**Figure 1:**
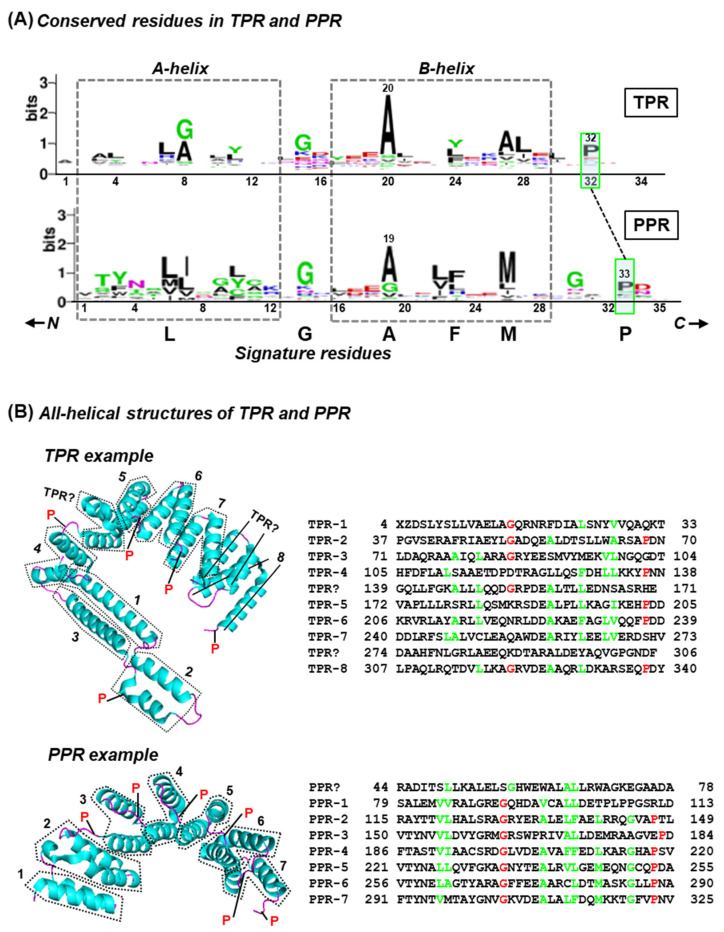
Primary and three-dimensional (3D) structural commonalities of TPR and PPR. The amino acids are written in standard single-letter codes; Different colors were used to provide easy correspondence between the sequence and the structure display, as appropriate. (**A**) WebLogo plot [12,13,14] derived from a comprehensive collection of 1137 TPRs and 22,999 PPRs (respectively, 34-aa and 35-aa long) is shown. The residue colors were auto-chosen by the program and can be ignored for this paper. In the WebLogo plot, the height of a letter indicates its relative occurrence compared to other letters at that position. This also displays the conservation of the ‘signature’ residues in corresponding positions; TPR-A20, for example, is essentially PPR-A19. Six predominantly conserved residues (L, G, A, F, M, P) are shown at the bottom. Importantly, the sequences of the TPR and PPR are virtually indistinguishable. The amino and carboxy terminal directions are indicated by N and C, respectively, with arrowheads. The two conserved helices, A and B, are approximately marked by dotted rectangles. The one additional residue in PPR over TPR seems to be inserted in the relatively variable sequence downstream of the B helix, as indicated by the shift in the strongly conserved P32 in TPR to P33 in PPR (boxed in transparent green color), when the other conserved residues of the two repeats are aligned. (**B**) Experimentally determined 3D structures of representative TPR (PDB 7RQF) and PPR (PDB 4M57) are presented in PyMol display [15] on the left. The alpha-helices are in teal color; connecting loop regions are in red. The repeats are numbered arbitrarily for this presentation, but the same numbers are used in the primary and 3D structures. Two regions in the TPR protein and one in the PPR, denoted by a question mark, are bihelical and also resemble TPR/PPR 3D structures, but they are missing several signature residues of TPR/PPR. The X and Z in TPR-1 are Met and Glu, respectively, but the X-ray diffraction did not reveal their structure, perhaps due to their terminal location in the polypeptide. The most predominant residues, determined by WebLogo plot, are colored in green here, while the helix-bender Gs and helix-breaker Ps are in red. For space constraints, only the Ps are indicated in the 3D structures. Each PPR bi-helix is demarcated with a dotted enclosure. These structures were essentially identical to those predicted by AlphaFold 3, especially in the high-confidence helical regions (pLDDT mostly >90; in a few areas, 70–90).

The tricopeptide repeats are named by the number of amino acids that each repeat unit contains, as exemplified by the two earliest families known, namely tetratricopeptide repeat and pentatricopeptide repeat (TPR and PPR), composed of 34-aa and 35-aa units, respectively [1,2,3,4,5,6]. In this nomenclature, tricopeptide is a 30-aa-long peptide, and additional amino acids are added as a suffix; thus, 4 (tetra) + 30 (trico) is a tetratricopeptide repeat (TPR), which is 34-aa long. Similarly, 5 (penta) + 30 (trico) is a pentatricopeptide repeat (PPR), that is 35-aa long. The family has been expanded by the recent recognition of larger tricopeptide repeats, namely heptatricopeptide (37-aa) repeat (HPR) and octatricopeptide repeat (OPR). Each unit repeat contains two helices that are ~14-aa long and designated A and B; in each repeat, specific positions contain residues that are predominant, though not invariant (Figure 1). TPR and PPR, the best studied of these repeats, occur in multiple numbers in biological polypeptides and are connected by linkers or spacers. In the past few years, we and others have analyzed the TPRs and PPRs, including their higher-order structure and the spacing of the repeat motifs [7,8,9,10,11,12,13]. Collectively, these studies have shown that both repeats are highly degenerate in primary structure, such that they are nearly impossible to recognize in a polypeptide without the use of computer algorithms. Analysis of a total of ~24,000 PPR and TPR proteins in extant genomes also revealed an enormous diversity in the number of repeats as well as the spacer (linker) sequence between two consecutive repeats.

These bioinformatic studies have been aided by experimentally determined three-dimensional (3D) structures of several TPR domains, and more are being determined [16,17,18], including those of the IFIT family proteins, such as IFIT5 (also known as interferon-stimulated gene 58 or ISG58) [18,19]. It is an all-helical protein comprising 24 helices, of which ~11 are tandem TPRs and the rest, TPR-like, i.e., missing some of the signature residues [19]. The difficulties of demarcating a PPR or TPR motif also stem from the uncertain termini of each motif, particularly when the sequences are highly degenerate. These problems were found to be more acute in HPR and OPR mentioned above. As a result, a unified perspective of all tricopeptide repeats has not been proposed yet. Similarly, the continuum of repeat lengths or a possible common origin of the TPR/PPR/OPR/HPR motifs has remained unaddressed. This review has used various similarity algorithms, incorporating AI, Monte Carlo simulations, as well as 3D-structure predictions and visual inspections to fathom the difficulty inherent in the number-based nomenclature of these degenerate repeats and focus on the uniformity among them.

Since the TPR and PPR repeats have been known for some time, several reviews have been devoted to them [2,9,10]; thus, these two repeats will be discussed briefly, while the other tricopeptide repeats will be presented in greater detail.

## 2. Consensus Amino Acid Sequence and All-Helical Structures of TPR and PPR

Several amino acid residues are highly conserved in both TPR and PPR, often serving as the signature residues of the motifs, as shown here in a simplified WebLogo presentation (Figure 1). In contrast to globular domains, repeat domains consisting of tricopeptides have regular, modular, and linearly arrayed structures, which also generate ordered, repeating helix-turn-helix structures in 3D, amenable to thermodynamic and biophysical analysis.

The rationale behind choosing the two structural examples (Figure 1B) is as follows. Due to technical difficulties, mentioned in Section 6 (Limitations), crystal structures have been solved for only a handful of TPR/PPR and deposited in the RCSB PDB bank, many of which were from synthetic idealized repeats. The PDB structures in Figure 1B were chosen for presentation as they are from naturally occurring proteins, likely with a biological function. While 7RQF is the crystal structure of LbcA (lipoprotein binding partner of CtpA) from the bacterium, *Pseudomonas aeruginosa*, 4M57 is the crystal structure of the plant PPR protein, PPR10, from maize.

When the locations of the signature residues in TPR and PPR (Figure 1) were adjusted for the length difference of one residue between TPR and PPR, they could be well aligned, as shown [13]. Both repeats are bi-helical, forming two helices, A and B [11]. When incorporated in a polypeptide, Pro is a unique amino acid as its nitrogen is not bound to any hydrogen, and thus, it cannot act as a hydrogen bond donor. As a result, its presence in a helix breaks the continuity of the helix [20,21,22]. In TPR, this role is served by P32; although its apparent counterpart in PPR is P33, it is far from the base of the B helix, and thus unlikely to act as a breaker for the B helix. At the same time, Gly30 appears as a predominant amino acid in PPR. Although Gly is not a helix-breaker, its side-chain H is the smallest among amino acids, which distorts the helix, visible as a bend or kink [22]. Thus, it can be concluded that this Gly, along with the neighboring residues, is primarily responsible for B helix distortion, which is finally broken by the downstream P33. A similar mechanism, i.e., distortion of various degrees, may be operative in other motifs that are also missing this Pro, such as TPR-1, -3, -7, and the two TPR-like and one PPR-like helices.

In recognizing a tricopeptide repeat, some degeneracy of sequence conservation is accepted, including the absence of a few signature residues, since these repeats often function by the interactions of amino-acid side chains of similar size and charge. The sequence diversity is evident in the TPR and PPR examples shown here (Figure 1). For example, among the four major hydrophobic amino acids with large side chains (M, I, L, V), the WebLogo reveals that L and I are of the highest occurrence in positions 6 and 7 of PPR; however, they are often substituted by other hydrophobic residues that are displayed in smaller letters. Bihelices (Figure 1) that maintain the continuity of the superhelix but lack the signature residues are often found among all tricopeptides and likely represent greater degeneracy in helical repeats that are innately degenerate. As indicated here and in Table 1, these deviants still retain the side chains for stabilizing interactions with neighboring helices, which may have prevented their loss during evolution.

## 3. Subclasses of PPR

### 3.1. Noncanonical Lengths and Diverse Arrangements of PPR Motifs

Noncanonical PPR motifs and their unusual arrangements were first noted during mining of the *Arabidopsis thaliana* genome and the termed Combinatorial and Molecular Protein (AtPCMP) family [1]. Further independent studies extended to the length of the motifs and their arrangement in a polypeptide, which led to the proposition of multiple PPR subclasses [13,23,24,25]. The lengths of the linker sequences joining them were also found to be variable [13].

**Figure 2:**
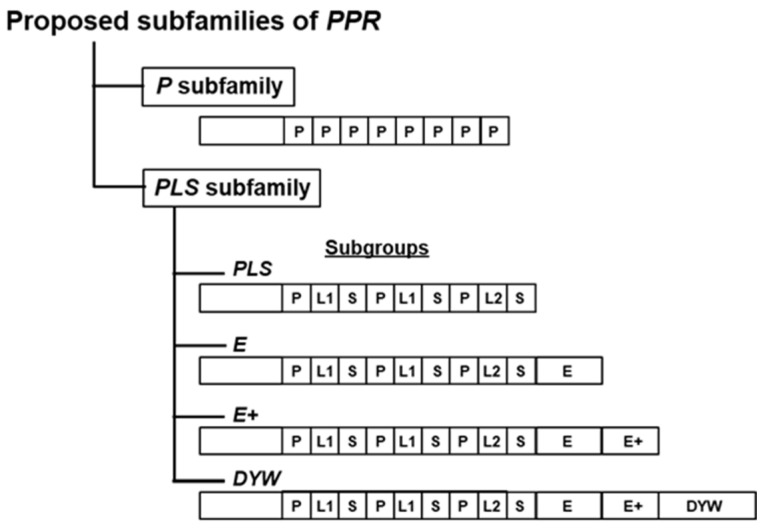
Schematic diagram of the PLS nomenclature system. It starts with two large subfamily names, P and PLS, as shown. The PLS subfamily is further classified into the PLS subgroup, E subgroup, E+ subgroup, and DYW subgroup. P stands for pentatricopeptide, L for Long, and S for Short. L and S are PPR-like but miss several conserved PPR residues. The subgroup named DYW contains a conserved C-terminal area that encodes a peptide stretch containing conserved D, Y, and W residues, hence its name. In reality, the actual protein sequences that occur in various species are much more complex, as they may contain a diverse number of each repeat type, which may also differ from this idealized arrangement. The PLS subgroup, for example, generally contains many repeats of the P-L1-S module with degenerate sequences. For such reasons, it is also not known whether the length of the protein is predictive of the subgroup, for example, whether all DYW domain proteins are longer than the E proteins. The arrangement may also vary between different types of plants.

In recognition of the noncanonical or deviant PPR repeats, two subfamilies were proposed and designated P and PLS (Figure 1 of Shikanai, 2006) [26]. The authentic PPR proteins, in which the PPR repeats are relatively highly conserved, were classified into the P subfamily. This immaculate scenario was rattled by the observation that many repeats encoded in the same species deviated from the consensus as well as the 35-aa length [1,13,23]. A new subfamily, designated PLS, was created to accommodate these PPR-like repeats, discovered exclusively in plants. This subfamily also included PPR proteins with C-terminal extensions that contained additional motifs, namely E, E+, and DYW, as shown in Figure 2.

The groups were further divided into subgroups S (for short) and L (for long). In the PLS subfamily, the tandem arrays of PPR or PPR-like motifs are usually followed by several conserved motifs, namely E, E+, and DYW (named after its conserved Asp, Tyr, Trp residues), arranged in this order. Based on these C-terminal motifs, the PLS subfamily is divided into four subgroups: PLS (without any C-terminal motif), E (with E), E+ (with E and E+), and DYW (with E, E+, and DYW) (Figure 2). Of note, some authors have identified these motifs somewhat differently and used variations of this nomenclature, such as E2 instead of E [27]. Although members of the P subfamily are widely distributed in eukaryotes, the PLS subfamily occurs only in plants [5]. Many PPR proteins that have been studied in detail were found to serve specialized functions [28], as discussed briefly below.

PPR proteins recognize specific RNA sequences and thereby regulate cardinal steps of RNA metabolism, such as RNA-base editing (mainly C to U and less frequently, U to C), splicing, stability, cleavage, and translation initiation [26,29,30,31,32,33,34,35,36]. Since RNA editing is prevalent in plant organelles, namely chloroplasts and mitochondria, plant PPR proteins play a major role in organelle biogenesis and function, impacting essential functions, such as photosynthesis, respiration, and responses to the environment. Although encoded in the nuclear genome, most of the PPR proteins in a plant cell, which often number in the hundreds, encode plastid- or mitochondria-targeting signals at their N-terminus (Figure 2) and localize to these organelles [23]. A mounting body of complementary evidence has shown that mutations in specific PPR proteins, either occurring naturally or introduced by directed mutation, exhibit phenotypes related to loss of RNA editing at specific sites in the organellar RNA.

As more genomes of the plant kingdom were sequenced, species-specific similarity and differences in PPR sequences emerged, adding further complexity to the PPR family [5,24,32,37,38,39,40]. Comparison of PPR sequences in *Arabidopsis*, rice (*Oryza sativa*), and *Physcomitrella patens* (a moss), for example, showed similarity and dissimilarity between orthologs as well as in the intron content of the PPR genes, as recently summarized [37,41]. Expectedly, these variations also added significant hurdles in determining the start and end of a repeat unit [13]. It is, therefore, reasonable to assume that a large number of tricopeptide repeats have remained unrecognized, as recently noted for *Chlamydomonas reinhardtii*, a single-cell green algae [6,39]; when identified, these sequences may extend the diversity.

### 3.2. Function of the Noncanonical PPR-like Motifs

While the P subfamily (Figure 2) essentially comprises the canonical or true PPR (presented in Figure 1) that is ubiquitous in the eukaryotic kingdom, the PLS-class proteins are mainly restricted to land plants [5] and, therefore, appear to be more recent in evolution. Thus, any unique function of the PLS proteins is likely related to the biochemical processes in the land plants, but this is not very helpful in understanding their differences. On the other hand, considerable progress has been made in mapping the interactive sequences of PLS proteins and their RNA substrates. Essentially all PPR proteins modulate RNA metabolism, but with individual specificity [42]. As a rule, the P segments of PLS proteins are involved in passive binding to non-coding RNA sequences, by which they facilitate intron splicing, protect the RNA from nucleases, and ensure proper folding of the RNA to allow translation [40,43]. In contrast, those containing E and DYW segments are associated with post-transcriptional RNA-base editing (mainly C-to-U, sometimes U-to-C) [24,32,43,44,45,46].

Several structure–function relationship studies have mapped RNA–PPR interactions at the molecular level, a full discussion of which is beyond the scope of this review. Their salient features will be summarized here only to understand if the arcane sequence diversity of these repeats is related to their functional diversity. Much of this complexity is rooted in the fundamental principles of nucleic acid–protein interactions, in which the trans-protein is recruited to a cis-acting RNA sequence, thus conferring specificity of function. This is exemplified in the PLS proteins, whereby the PPR domain specifically binds to the 5′ sequence of the target cytidines; as a result, the DYW domain of the same polypeptide is properly positioned and catalyzes C-to-U deamination with its deaminase domain [27,45,47,48,49,50]. This allows discrimination between target and non-target sites. There are a few examples of DYW domains possessing site-specific endoribonuclease activity, the detailed mechanisms of which remain unknown [51,52]. To reiterate, the DYW domain, by itself, does not bind RNA; rather, its interaction with RNA is assisted by accessory proteins, including the PPR domain in *cis*, thus involving complex tripartite interactions [45,53].

### 3.3. PPR–RNA Interaction Code

Here, I will focus on direct PPR–RNA interactions that have been shown to follow sequence-dependent recognition codes, generally referred as ‘PPR–RNA interaction code’ [34,54].

**Figure 3:**
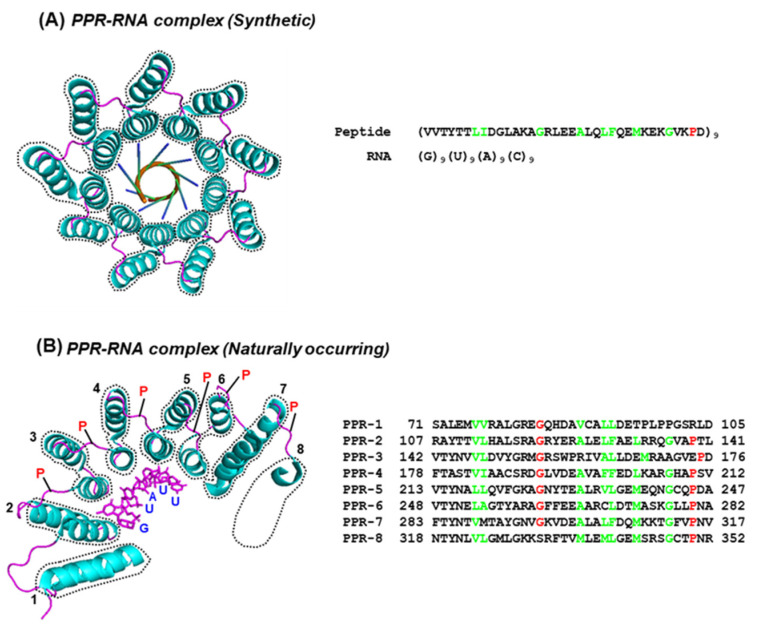
PPR–RNA interactions. The crystal structures of two PPR–RNA complexes are shown in PyMol [15] representation; Different colors were used to provide easy correspondence between the sequence and the structure display, as appropriate. (**A**) A synthetic designer PPR consisting of a single representative PPR motif from PPR10 of Zea Mays, repeated nine times and complexed with a synthetic RNA (PDB 6EEN) [55], which is also repeated nine times. This is the top view of a complete superhelical turn; hence, only the corresponding segment of the RNA is shown. The amino acid and nucleotide sequences are written on the right side. The RNA is magenta colored; other colors are the same as in Figure 1. (**B**) Naturally occurring pentatricopeptide repeat of PPR10 in complex with its cognate binder RNA in the cell, RpsJ (PDB 4M59, Chain A) [56]. Only six nucleotides of the 18-nt-long RpsJ sequence make contacts with the PPR, five of which (GUAUU) are shown here as representative. The full-length PPR10 contains at least 20 double-helical regions, including 18 PPRs, 8 of which are shown. The GUAUU binds to five helices of consecutive PPRs (numbered 1 through 5). As in panel (**A**), each PPR bi-helix is bordered with a dotted line. To conserve space, only the N-terminal portion of PPR-8 is shown because it is adjacent to the Pro that serves as a helix breaker for the preceding helix of PPR-7.

In this scenario, when a PPR domain straddles on the RNA, each repeat contacts a specific nucleotide (Figure 3).

**Figure 4:**
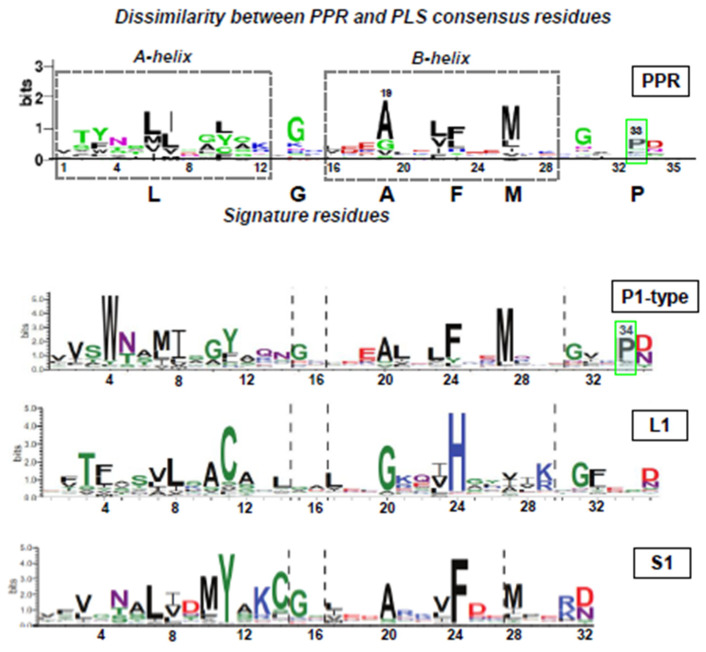
Dissimilarity between the PPR consensus and the PLS family sequences. The PPR WebLogo is from Figure 1A, which was adapted from Barik [13], and the others are from the pioneering large-scale study of Sun et al. [28] in compliance with the CC-BY-NC-ND reuse option. For such reasons, it is also not known whether the length of the protein is predictive of the subgroup, for example, whether all DYW domain proteins are longer than the E proteins. The arrangement may also vary between different types of plants. The default WebLogo colors have been used.

Note that the PPRs in the designer structure (Figure 3A), the repeats, are all identical in sequence, which is also reflected in the perfectly symmetrical superhelix. In contrast, the superhelix in the PPR10–RpsJ complex (Figure 3B) describes the symmetrical centering of the repeat RNA, which contrasts the structural variation in the natural PPR–RNA complex in panel A, commensurate with the requirement of the PPR to bind the varying RNA sequences. The deviation from canonical PPR is all the more pronounced in the PLS subfamily, as noted by pioneering studies of several groups, notably Ian Small and co-workers [24,26]. To illustrate this, four representative PPR-related WebLogos are presented (Figure 4) from previous collections: the canonical PPR (repeated from Figure 1A for comparison), and three sequences from the PLS subfamily, viz., P-type, L1-type, and the S1-type.

The differences in the PLS subfamily sequences from true PPR (P subfamily) are obvious in their lengths, the shifted positions of the apparently conserved signature residues when present, and the different conserved residues (Figure 4). Attempts were made to find the optimal sequence similarity in various ways [5,26,28,54,57], including our own attempts, as shown here, in which the helix boundaries were aligned, since the bihelical structure is a fundamental feature of all tricopeptide repeats and is especially useful as a landmark when the lengths are variable. As shown (Figure 4), helix-based alignment threw the numbers off the alignment as well as changed the scale to some extent; however, despite these compromises, no positional commonality in the signature residues was apparent among the three PLS subtypes. There were a few minor similarities between P1 and L1, such as W4 and F4 (acceptable because W and F are both aromatic). Regarding P1 and S1, once their length difference is accepted, their WebLogo patterns appear relatively similar, including the positions of the conserved residues, such as N5, Y11, G15, A20, F24, and M28. Notable absentees in some motifs included the G31 of P1 and L1 in S1, and the helix-terminating PPR-P34 in both L1 and S1. Interestingly, the DN dipeptide is the only major WebLogo residue present in the C-terminus of all subtypes. These difficulties of alignment in the PLS family starkly contrast with the ease of alignment between TPRs and PPRs (Figure 1) due to their multiple homologous residues. Some E motifs near the C-terminus resemble PPR motifs in their nucleotide-binding specificity [58] but are not discussed here for the sake of brevity.

An important outcome of these sequence analyses was their translation into functional differences in the cell, which encouraged cracking of the PPR–RNA interaction code by multiple research laboratories at the atomic level. Such studies generally comprised computational analyses of nucleotide–amino acid interaction using various RNA substrates, crystallography of PPR motifs in PPR as well as PLS proteins, screening of mutants that are defective in RNA editing, reverse-genetic studies of specific mutations and domain-swapping on the editing function, and artificial evolution of target RNA by SELEX (Systematic Evolution of Ligands by Exponential Enrichment) [27,30,45,54,59,60,61]. For brevity, a few key rules of the interaction code are presented here: (i) All three major PLS motifs (L1, L2, S) selectively contact nucleotides in the target RNA, as mentioned earlier; (ii) Certain amino acids play a greater role in target RNA identity; (iii) Not unexpectedly, the final choice of the RNA editing site is determined by the total interactions of multiple interacting motifs, e.g., L1, L2, and S; in other words, the inclusion of all three motifs improves the success rate of target prediction. These results allowed prediction of RNA editing sites by a given PPR type and recoding of a PPR protein to bind new RNA sequences [54,62,63], reciprocally validating the computations.

Overall, however, the predictions are still not perfect, and some failures have been noted, indicating the existence of other contributors that need to be included [53,54,64,65]. For example, a defect in an accessory factor target site in a plant variety of PPR may be compensated by another PPR protein, as has been documented for the Mitochondrial Editing Factor of *A. thaliana* [66]. It can be conjectured that the complexity of interactions may serve as an advantage in the proper context, such as the multiple PPR repeats may allow gaps in contacting certain RNA, thus allowing them to target a larger repertoire of RNA, perhaps with the help of RNA-specific accessory proteins [30,59,62]. The RNA repertoire is likely to be even broader for the PLS proteins because of the greater diversity of their repeats, which may have ensured their evolution in interaction with the larger and more complex RNA, while the more uniform PPR proteins were delegated to simpler and shorter RNA targets. In other words, the PPR motifs in general, including the ones in the PLS subfamily, must have co-evolved with their cognate RNA ligands.

## 4. Heptatricopeptide and Octatricopeptide Repeats

Considered members of the tricopeptide family, the octapeptide and heptatricopeptide repeats (OPR, PPR) are, respectively, 38-mer and 37-mer long and are much rarer than the PPR/PLS repeats. Unlike PPR, which is more abundant in plants than in algae [1,5,38,67,68,69], the octatricopeptide repeat (OPR) is prevalent in algae, such as *Chlamydomonas reinhardtii*, a unicellular alga that is capable of photosynthesis [70]. Interestingly, the HPR proteins were reported from a multistep, reiterated search that was both intuitive and serendipitous [71,72]. The full search is too elaborate to describe here in detail, but interestingly, the authors started *C. reinhardtii* OPR sequences as a query and performed a similarity search in the genome of *Plasmodium* spp., unicellular parasites of the Apicomplexan family. The sequences were confirmed to have homologs in two closely related species, *P. falciparum* and *P. berghei*, and later in *P. vivax* and *P. cynomolgi* (our unpublished data) [73], which is considered an accepted criterion of an authentic gene. Thus, the very discovery of HPR underscored their closeness to OPR and distance from TPR and PPR.

### 4.1. Heptatricopeptide Repeat (HPR)

As mentioned earlier, Hillebrand et al., in 2018 [72] used a combination of bioinformatics and biochemical approaches to identify a new class of 37-aa repeat that they named heptatricopeptide repeat (HPR). The HPR proteins were initially found in the Apicomplexa mitochondria, but subsequent phylogenetic search identified them in a wide variety of other eukaryotic taxa. These proteins bound RNA, but not DNA, and many of them contained a RAP domain (an acronym for ‘RNA-binding domain abundant in Apicomplexans’) near the C-terminus [72,73,74].

#### 4.1.1. *P. berghei* HPR Proteins

**Figure 5:**
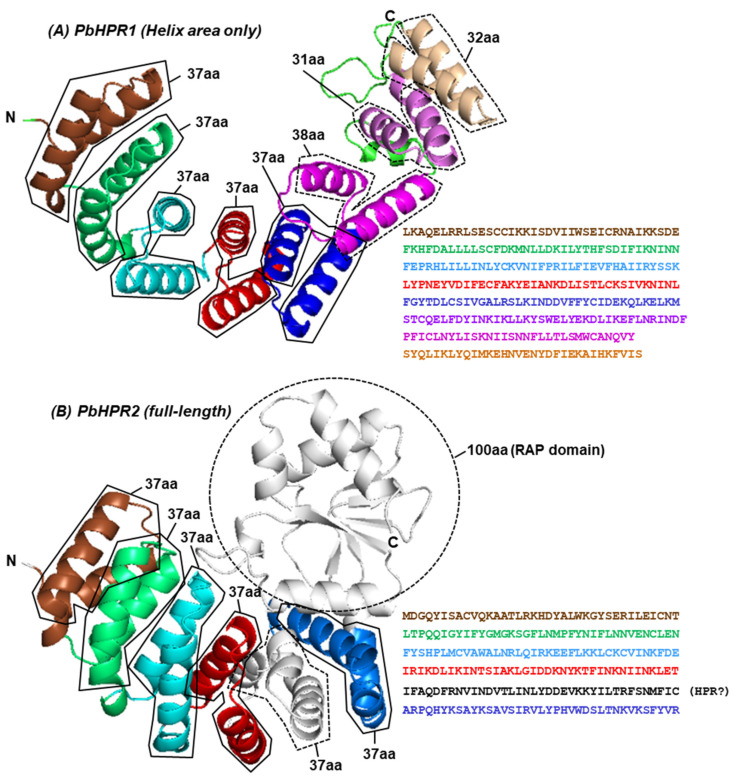
Predicted structures of two putative HPRs in the protozoan *Plasmodium berghei* (strain Anka): PBANKA_051680 (Protein XP_034420409.1; PbHPR1) and PBANKA_051380 (Protein XP_034420379.1; PbHPR2) [72]. The three-dimensional structures were determined by the AlphaFold 3 server (which uses AlphaFold 3, the latest version; accessed at https://alphafoldserver.com/, 30 October 2025), imported as a PDB file, and then displayed with PyMol [15]. In both panels (**A**,**B**), all helix pairs are boxed and individually colored, starting with brown at the N-terminal end. The amino acid sequences of each repeat, shown on the right, match the color in the structure. The repeats were aligned using the multiple sequence alignment (MSA) programs Clustal Omega, MAFFT, and T-Coffee [75]; their consensus was determined by visual inspection and presented here. These MSAs were all obtained at the EMBL-EBI site (https://www.ebi.ac.uk/Tools/msa/), as described previously, and were accessed 30 October 2025 [12]. Note that AlphaFold and Interpro both use an AI-powered deep learning model developed by Google DeepMind (London, UK). Alphafold structure predictions here and elsewhere in this review were conducted with AlphaFold 3 on the server at alphafold.com [76], in which the single-letter amino acid sequences were entered in FASTA format. The pLDDT (predicted Local Distance Difference Test) score in AlphaFold is a per-residue confidence score that indicates how confident the model is about the accuracy of the predicted atomic coordinates of that residue. A pLDDT score ranges from 0 to 100, with higher scores indicating higher confidence. In all AlphaFold predictions conducted in this review, the helical areas of the tricopeptide regions exhibited high confidence in the 70–90 pLDDT range, and these are also the areas presented here.

No experimentally determined structure of HPR or OPR proteins has been reported to date. Hillebrand et al. [72] reported two *P. berghei* HPR-domain proteins in relative detail and predicted their structure using I-TASSER, which suggested that both domains consisted of a series of bihelical HPR units [72]; I have confirmed and extended the structure using AlphaFold 3, the AI-based program (Figure 5).

As shown (Figure 5), in PbHPR1 (Panel A), the helix-rich 37-aa HPR area is followed by 38-aa, 31-aa, and 32-aa motifs that are also bihelical. In the PbHPR2 protein (Figure 5B), an approximately 100-aa bundle encompasses a putative RAP domain and several other domains, according to Interpro-N, an AI-powered deep learning model developed by Google DeepMind (accessed on 30 October 2025, at the EBI site: https://www.ebi.ac.uk/interpro/). The RAP area is colored gray. Interestingly, the continuity of the HPR vortex in this protein is disrupted by a 37-aa stretch that is a broken bi-helix (also in gray). This sequence was not considered an HPR [72]; nonetheless, as presented in Table 1, the side chains of the small helices interact with various side chains of the proximal HPR helices (red and blue). These contacting side chains are not shown in PyMol [15] for easy viewing of the structure. In transfected *P. berghei*, the mCherry-tagged proteins appeared to colocalize in the mitochondria [72]. Lastly, in affinity-tag pull-down experiments, recombinantly expressed MBP-fusion PbHPR1 protein bound to RNA transcribed from cloned *P. berghei* mitochondrial DNA in vitro, although the RNA-binding domain(s) of the protein or the interacting nucleotides were not mapped.

The free energies of interaction were obtained at the Amino Acid Interaction Web Server (http://bioinfo.uochb.cas.cz/INTAA/; accessed on 30 October 2025), which utilizes an Interaction Energy Matrix (IEM) and lists contacts made by each amino acid and its interaction energies [77]. The most stabilizing interactions are also important for stability and correct protein folding. Here, a baseline of −4 (negative 4) was chosen to keep the table in a manageable size; in other words, the major contacts with energy values of magnitude >4 were collected and tabulated. The choice of −4 as baseline did not affect the conclusions, since the majority of the interactions were much stronger (e.g., −22.26 kJ) in the last line, which is the strongest interaction in this Table. It is to be emphasized that, useful as they are, the energy values were computed on predicted structures that need experimental validation.

Table 1 lists the free energy of trans-interactions between the last HPR and the non-HPR helix that follows. In PbHPR1, this interaction occurs between HPR helix F198–GYTD, KELKM-234, and the next two bihelices (235—272: 37-aa, 294—314: 31-aa). In PbHPR2, it is among the last HPR 170-RIKD---KLET-205 [72], and the next fragmented helical part (206—242).

The roles of the non-HPR helices, i.e., the octa-, mono- and di-tricopeptide motifs in PbHPR1 (Figure 5A) and the fragmented 37-aa helix in PbHPR2 (Figure 5B) are unknown; however, in studies of TPR, others and I previously suggested that these helices, which do not conform to the repeat sequence, nevertheless make contacts with the repeat helix that is within atomic distance, as judged by the negative free energy of interaction between their side chains [11,12]. It was concluded that these helices most likely contribute to the overall structure of the protein and perhaps also facilitate its solvation. I conducted a similar analysis for both proteins. In HPR1, I focused on multi-helix interactions among the amino acid side chains of the last HPR helix [72] (blue in Figure 5A) and the next two non-HPR helices (38-aa, red; and 31-aa, pink). The interaction energies were determined using INTAA, as described. The results (Table 1) clearly revealed strong stabilizing interactions among all three helices, including the 31-aa one. For example, in the HPR helix, Asp225 interacts solely with Tyr257 of the 37-aa non-HPR helix with a strong stabilizing energy of −13.79 kJ. Thus, a helix can provide stability to a repeat area even if it is neither a part of the HPR superhelical vortex nor a clean bihelical segment, such as a repeat. In this respect, the HPR resembles TPR and PPR, in which essentially any helix functions as the energetically stabilizing or “solvation helix” [11,12].

Interestingly, *P. falciparum* (Pf) proteins often contain long homopolymeric stretches of asparagine (N); this is due to the fact that the Pf genome is AT-rich and so are the Asn codons (AAU, AAC). However, as noted previously [72], the N runs do not occur inside an HPR helix. This may not be a specific feature of the HPR helix but may simply reflect the fact that Asn is generally disfavored in the interior of an α-helix, as its polar amide side chain can interfere with the helix’s main-chain hydrogen bonding.

#### 4.1.2. *T. gondii* HPR Proteins

**Figure 6:**
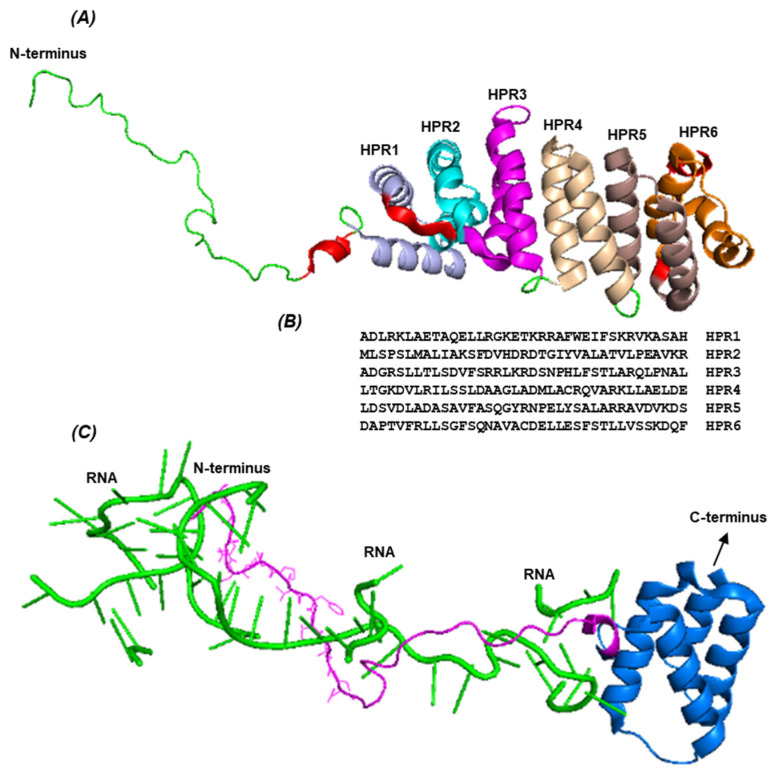
Portions of a mitoribosomal HPR protein of *T. gondii* ME49 (TgME49_309790) and its association with mitochondrial RNA fragments [78]. (**A**) HPR domain of the protein, located after the long and flexible N-terminus (Green). The six HPR bihelices are shown in six different colors. (**B**) Sequences of the HPR. (**C**) Within the mitoribosome, at least three RNA segments (green) are located in close proximity to the N-terminal end of the protein. Note that the same N-terminal end is also shown in Panel A, but in green; here, it is shown in pink so that the RNA can be shown in green. The four helices, shown in this panel, are also present in Panel A, but in different colors and orientations. For ease of viewing, all other mitoribosomal proteins have been hidden using the ‘Hide’ function of PyMol [15]; for the same reason, neither the protein residues nor the RNA nucleotides are labeled. These structures are all derived from the cryo-EM of the Tg mitoribosome [78].

The *T. gondii* genome codes for at least 25 HPR proteins [72,78], but perhaps more, since HPR sequences are difficult to recognize. Although the exact role of most tricopeptide repeats is not known, as stated for PbHPR1 and PbHPR2 above, *Apicomplexan* HPR proteins traffic to the mitochondria. Recently, a collaborative effort by multiple laboratories led to the cryo-EM structure of the *T. gondii* mitochondrial ribosome (mitoribosome) [78], which revealed the locations of several HPR proteins in it. Of these, the protein designated mL162 (TgME49_309790) was chosen, as it was determined essential by CRISPR/Cas9 knockout, although its precise function is unknown [79]. Its location near the apex of the mitoribosomal large subunit facilitated its viewing in the EM image and presentation in PyMOL (Figure 6).

The 3D structure of the Tg mitoribosome clearly offers a molecular treasure trove of multiple RNA-protein and protein–protein interactions of an HPR in a biological context. Only an example of it is provided here (Figure 6).

### 4.2. Octatricopeptide Repeat (OPR)

The OPR domains were known years before the HPR [67,70,80,81,82,83]. In fact, as indicated earlier, the identification and justification of HPR as a new class, different from OPR, required multiple reiterative bioinformatic operations and biochemical experiments [72], part of which served to eliminate the OPRs, underscoring their closeness. As further elaborated below, discussions of HPR and OPR often go together. Lastly, with all tricopeptide repeats, but more so with HPR and OPR, short gaps are sometimes allowed for the sake of alignment to an amino acid consensus.

While authenticating HPR as a new tricopeptide repeat, Hillebrand et al. plotted the WebLogo of a total of ~67 HPR proteins from three Apicomplexa parasites, viz., *P. falciparum*, *P. berghei*, and *Toxoplasma gondii*. The plot suggested Helix A and Helix B in each HPR motif with the following spacing: (4-aa)—(12-aa Helix A)—(4-aa linker)—(15-aa Helix B)—(2-aa), which is reminiscent of the general architecture of TPR and PPR [72]. These authors also collected 43 OPR motifs from *C. reinhardtii* and generated a WebLogo plot. Neither of them revealed a strict consensus amino acid sequence with a dominant amino acid in any one position [72]. In both HPR and OPR, hydrophobic residues, namely Leu and Ala, were prominent and distributed over the length of the motif; in OPR, two others, namely P22 and W26, were also prominent. This is in clear contrast to TPR and PPR, where several amino acids are clearly dominant, such as G15/14, A20/19, and the helix breaker P32/33 that defines the end of each repeat (Figure 1).

**Figure 7:**
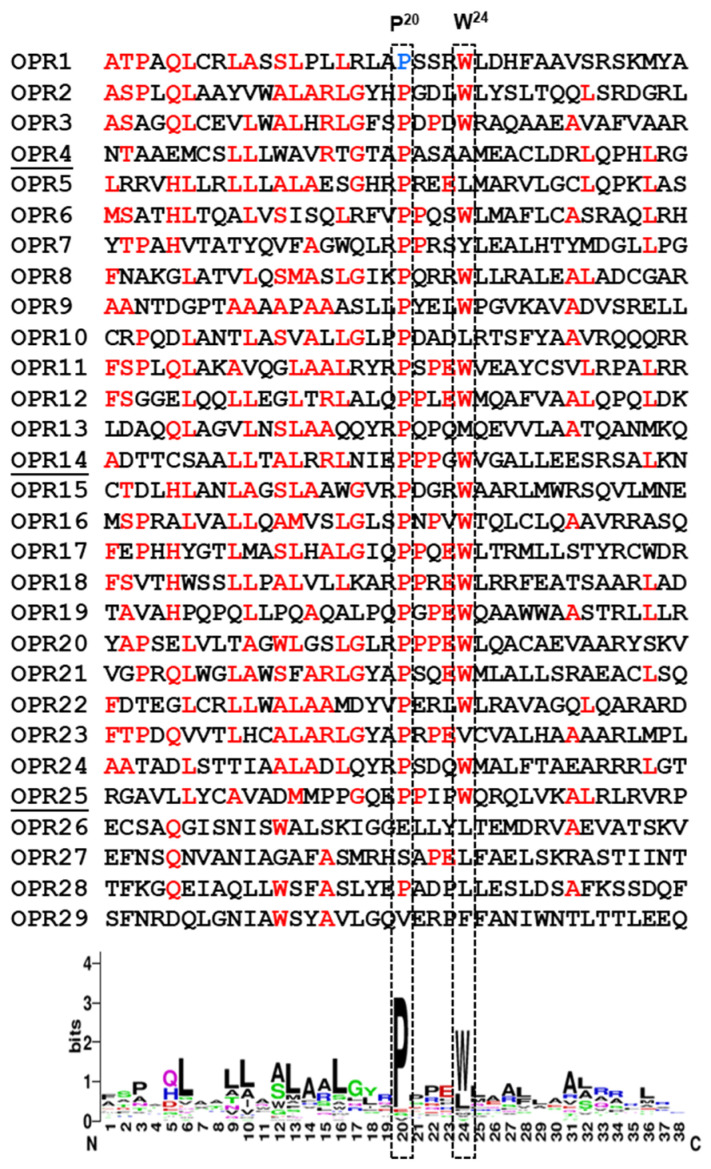
Representative OPR sequences (38-aa) from the proteins TDA1 (OPR1-9), TBC2 (OPR10-20), RAA1(OPR21-25), and RAP (OPR26-29), which contributed a total of 29 OPRs, as shown and numbered serially. The WebLogo plot is shown below, in which the dominant P20 and W24 are boxed with dashed lines. A few other residues that are prominent in the WebLogo are colored in red (e.g., Q5 and L6).

Since the OPR consensus has remained relatively under-investigated, I followed up on this lead to illustrate the OPR WebLogo, first using the OPR sequences from three authenticated proteins of *C. reinhardtii* chloroplast, viz., the translation factor, TDA1 (CCA62914.20), another translation factor, TBC2 (CAD20887.1), and a putative group II intron trans-splicing factor, RAA1 (CAE53330.1) (Figure 7). To this, I have added the *Arabidopsis* protein, RAP, the most definitive OPR protein reported in a land plant [67]. Of note, another putative OPR protein, named RAAT2, was identified by the GreenGenie program, version 2, and its predicted amino acid sequence showed two bihelical regions with significant similarity to consensus OPR WebLogo, including a degenerate PPPXW peptide. However, it was not included in the analysis here because neither the transcript nor the protein could be experimentally detected [84].

**Figure 8:**
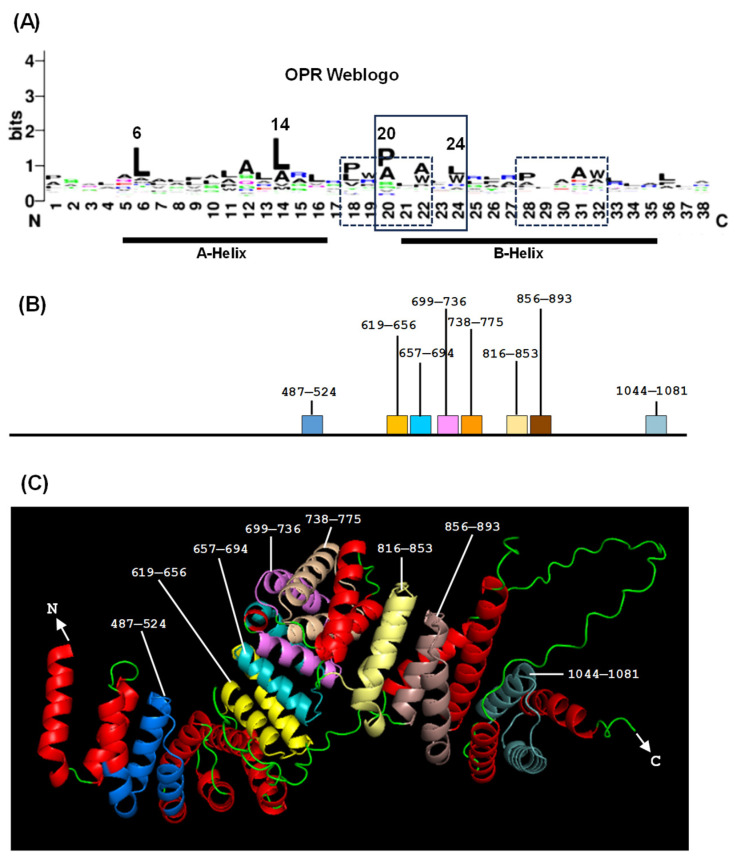
The prototype OPR domain of the *C. reinhardtii* protein, TBC2. (**A**) Recreated WebLogo of OPR sequences from all the *C. reinhardtii* OPR proteins (which includes TBC2), with the helices and the PPPEW motifs marked. (**B**) Schematic locations of the eight OPRs of TBC2 in amino acid numbers. (**C**) AlphaFold structure of the TBC2 OPR domain. Each OPR is individually colored, and the non-OPR helices are in red.

The residues P20 and W24, which are boxed here, are part of the PPPEW motif of OPR, which is discussed later in detail. This WebLogo pattern is very similar to that obtained previously by Hillebrand et al. using a larger cohort of all 150 OPR sequences from *C. reinhardtii* [72]. This list was generously shared by Professor Christian Schmitz-Linneweber [72], from which the family WebLogo was generated (Figure 8A). The A and B helices were marked by approximate consensus, but here, as well as in all other tricopeptide repeats, the boundaries of these helices vary from one repeat to another. The most conserved PPPEW motif referred to earlier is also marked. In addition, I have noted two other PPPEW-like motifs that are less conserved (Figure 8B). It is generally assumed that the OPR domain is composed of bihelical repeats, but no OPR structure has yet been solved. Here, I used AlphaFold 3 [76] to predict the 3D structure of the prototype *C. reinhardtii* protein, TBC2, to illustrate the repeat structure, accompanied by a location diagram of the eight OPR motifs on the polypeptide (Figure 8B,C).

The degeneracy of the PPPEW motif of OPR is worth noting. Although the most reliable consensus motif of OPR also sets the OPR apart from HPR, it does exhibit degeneracy, which was noticed in multiple studies early on [70,80,83], whereby the P in the first position and W were found to be the most conserved [83]. In the *C. reinhardtii* OPRs, for example, I have noted a large variety of deviations from the idealized PPPEW, such as loss of one or two P, loss of W (e.g., PQPQM, PGPRC, PPPAL), or (more commonly) replacement of the W with F, another aromatic residue (e.g., PSGRF, PEPAF, PPPAF, PSAAF). In nearly a quarter of the OPR motifs, the pentapeptide could not be recognized at all (e.g., PQDVSNVCWSLAALRVRPGVPLLQRLVARALAVRRRMK). Due to its unique structure with a large π-electron cloud on the fused aromatic rings, Trp in the PPPEW motif may have important roles in the OPR structure [85]. Nonetheless, the exact role of OPR, including the PPPEW motif, has remained unknown. Although HPR proteins are associated with RNA, neither their RNA interaction code nor the relationship with the accompanying RAP domains has been characterized.

## 5. Summary and Conclusions

To date, four classes of tricopeptide (thirty) repeats have been recognized and named by their lengths; they are tetra-, penta-, hepta-, and octa-tricopeptide, which are, respectively, 34-, 35-, 37-, and 38-aa long. As a rule, they are involved in interactions with proteins or nucleic acids, which translates into biological functions, such as allosteric changes in PP5 (a Ser/Thr phosphatase with an N-terminal TPR), various RNA processing and translational regulation by HPR, PPR, and OPR proteins. Despite this remarkable progress, our knowledge of tricopeptide repeats has remained woefully inadequate.

A fundamental question that arises in our search for tricopeptide repeats is how we recognize—and hence define—a “tricopeptide repeat”. As we realize now, the recognition minimally needs to take into account: (i) number of amino acids; (ii) bihelical structure; (iii) two or more occurrences in the same polypeptide. However, there are exceptions to each criterion, and this stems from the fact that the tricopeptide-repeat sequences are “degenerate”, which in practice means “unrecognizable”, since it precludes writing a computer program that will search for a particular string. As elaborated before, the problem is less acute for TPR and PPR, for which highly successful prediction toolkits with low false returns exist [86]. This is because the sequences and structures of TPR and PPR are relatively conserved; their signature amino acids are singularly predominant in WebLogo presentations; the helices are largely unbroken; and multiple repeats generate a geometric superhelix (Section 2 and Section 3). Because of these, false predictions are easily recognized and corrected. HPR and OPR present a more difficult situation, as there is no reliable recognition program; moreover, the lengths of the A and B-helices are also highly variable [87]. MEME and its several derivatives, which are different from AI, constitute a brilliant and popular repeat-finder suite for motif scan and comparison [88,89], and are used by almost everybody, but they are generally inadequate for this application. Motif search is ruled out, since HPR and OPR do not have a reliable motif. As discussed earlier (Section 4.2), the PPPEW motif of OPR is too short and degenerates, while HPR has no motif. Although MEME does not explicitly use AI, most sequence alignment and detection programs these days already use some form of AI and deep-learning models. Still, they are difficult to use and may not detect HPR and OPR motifs [88]. As a result, we currently rely on a small number of repeats that were examined, studied, and published by leading experts in bioinformatics and in protein structure and function, as described before (Section 4).

In revisiting the question in the Title, it is logical for one to wonder why some tricopeptide repeat numbers are missing, viz., 31, 32, 33, 36, and 39. This may be an illusion in that some of them are, in fact, not missing, but simply could not be given the honor of a tricopeptide name. As mentioned before, this group comprises a subclass designated L and S (Section 3), which were tricopeptide repeats in their architecture, but differed in length from the existing repeats, TPR and PPR. However, as suggested previously, these repeats can be viewed as a stepwise increment of length. Even TPR and PPR, the two founding members that differ in length by a single amino acid, contain the same signature amino acids [87]. While searching for TPR and PPR, I came across a large number of repeats that are shorter or longer than either of them, such as 32, 33, and 36, but they were called either TPR or PPR by subjectively defining the boundaries of the repeats to conform to either 34 or 35 [13]. As an example, consider a 33-aa and a 35-aa repeat in the same polypeptide connected by a 7-aa linker, as 33-7-35; but it can also be viewed as 34-6-35, by pushing the C-terminal end of the 33-aa repeat one amino acid further down, so that 33 becomes 34 and the 7-aa linker, 6-aa. Given the permissible variations in linker length [13], this will be considered acceptable. As a result, the 33-aa (tritricopeptide) repeat would remain unrecognized as such and construed as a TPR. Subsequent discovery of a functional distinction is unlikely to correct the name. I also showed that even very small size classes, viz., 26- 27- and 28-aa, contained the same signature residues as the longer members. Often, the increased length is due to a longer B helix, which pushed the invariant P32/33 gradually to P34, P35, P36, P37, and so on, eating into the following linker. However, the 36 was not recognized as a hexatricopeptide. The subjective aspect of tricopeptide recognition also applies to the decision of where the repeats start and end. Since 36 is not recognized as a named tricopeptide, the trend is to add a neighboring amino acid to make it a heptatricopeptide. I conclude that the overall helicity and the presence of critical amino acid residues are more important for the biological function of the tricopeptide repeats than the exact length. A unified view of the repeats may also help in predicting functions of newly discovered repeat proteins regardless of their length. Nonetheless, the length-based name remains important since it serves as a moniker for referral and recognition, if nothing else. With apology to Mr. Gulielmus Shakspere, a rose by any other name would, of course, smell as sweet, but changing it to *mihkokwaniy* will make it unrecognizable, except to those who speak Plain Cree.

## 6. Limitations of This Study

This section describes not only the major limitations inherent in this review but also the potential benefits that may accrue from them. Due to the general paucity of experimental data, nearly all the higher-order structures of tricopeptides presented here are based on theoretical algorithms, mainly AlphaFold. However, all structure prediction programs, especially AlphaFold, display their high-confidence and low-confidence areas. Fortunately, the helical areas are generally of highest confidence (>90%), and they also account for most of the tricopeptide repeat sequence. Nevertheless, experimental verification of AlphaFold structures is still necessary, especially for low-confidence helix-linker transition areas or in complex biological contexts such as multiprotein interactions, all of which may apply to tricopeptides. As mentioned earlier, while most predicted helices are of “very high” AI confidence (pLDDT > 90), several are designated just “confident” (90 > pLDDT > 70). A low-confidence helix or even a very-low-confidence structure of AI may lead to the discovery of a novel class of structural repeat motif.

Primarily due to their repeat nature and flexible structures, which are responsible for their diverse function, the tricopeptides tend to defy crystallization. The membrane resident repeats, such as the algal OPR, are also difficult to solubilize. Thus, a definitive understanding of their role in the living cell must await overcoming these hurdles. A largely overlooked aspect of AlphaFold is that it predicts several alternative structures of each protein (often called “recycles” or “models”) to account for the uncertainty in the prediction process. It is generally assumed that these structures are metastable and only one of them forms in the cell. However, the protein may adopt these structures under appropriate biological conditions and even switch between them as the conditions change. Such alterations in protein structure may form the basis of multifunctional proteins, also known as “moonlighting” proteins [90].

Although I have strived to provide a comprehensive treatise on all possible tricopeptides, this class as a whole maintains its variability in myriad ways, such as repeat number, primary as well as tertiary structures, and biological function. In an RNA-binding tricopeptide repeat, the RNA ligand itself adds further macromolecular complexity and diversity. It was not possible here to delve into this functional complexity, but relevant reviews and research articles by us and others have been cited, which offer such details.

## 7. Questions for the Future

Here, a few specific directions of future research in this area are suggested, framed in the form of questions and sometimes, answers. It is hoped that they will attract researchers of all levels to this exciting area of protein structure.

(i) Can we develop better computational detection algorithms, perhaps using machine learning, to find the difficult tricopeptide repeats, namely HPR, OPR, and the 31–33 or 36-residue repeats, in the protein databases? Machine learning may help here.
(ii) Once detected, how can we definitively determine whether these repeats exist as functional entities?
(iii) Can we obtain more experimental structures by cryo-EM [78], given the difficulty in crystallization of these proteins, to validate the predictions?
(iv) Can we venture into structural biology of greater difficulty, such as determining the structure of a protist mitochondrial HPR protein or an algal OPR protein in complex with RNA? For example, we can inquire if the HPR proteins function as ribosomal assembly factors in Apicomplexan parasites, as hinted by the *T. gondii* mitoribosome study [78].
(v) There are also many evolutionary questions to be asked; for example, did the algal OPR motifs evolve separately from land-plant PPRs, or can they be traced to a common ancestor [1,2,6]?

## Figures and Tables

**Table 1:** Energy of TPR–TCH interhelical interaction.

HPR (Protein) Name	Residue	Interacting Residue(s)	Interaction of HPR of Non-HPR Helixenergy (kJ/mol)
PbHPR1 (XP_034420409.1)	Asp225	Tyr257	−13.79
	Gln228	Tyr257	−16.16
		Leu203	−5.82
	Lys294	Tyr290	−9.33
	Ile296	Leu266	−6.76
	Ile297	His339	−7.34
		Leu302	−5.45
	Ser298	Asn270	−16.22
	Phe301	Leu305	−6.08
	Leu302	Ile297	−5.45
	Thr304	Phe272	−5.16
	Leu305	Phe301	−6.08
		Phe285	−5.58
		Trp308	−5.27
	Trp308	Gln312	−12.25
PbHPR2 (XP_034420379.1)	Asn196	Asp225	−25.66
		Val228	−4.65
		Tyr224	−4.05
	Asn198	Tyr224	−6.42
		Val228	−4.87
	Ile200	Tyr231	−10.98
		Val228	−4.25
	Leu203	Tyr231	−5.81
		Ile206	−4.24
		Val214	−3.89
	Glu204	Arg235	−22.26

## Data Availability

The original contributions presented in this study are included in the article. Further inquiries can be directed to the corresponding author.

## Abbreviations

The following abbreviations are used in this manuscript:

AI	Artificial intelligence
PPR	Pentatricopeptide repeat
TPR	Tetratricopeptide repeat
HPR	Heptatricopeptide repeat
OPR	Octatricopeptide repeat
3D	Three-dimensional
PDB	Protein Data Bank
RAP	RNA-binding domain abundant in Apicomplexans

## References

[1] Aubourg S., Boudet N., Kreis M., Lecharny A. (2000). In *Arabidopsis thaliana*, 1% of the genome codes for a novel protein family unique to plants. Plant Mol. Biol..

[2] Small I.D., Peeters N. (2000). The PPR motif—A TPR-related motif prevalent in plant organellar proteins. Trends Biochem. Sci..

[3] Pazour G.J., Dickert B.L., Vucica Y., Seeley E.S., Rosenbaum J.L., Witman G.B., Cole D.G. (2000). *Chlamydomonas* IFT 88 and its mouse homologue, polycystic kidney disease gene Tg737, are required for assembly of cilia and flagella. J. Cell Biol..

[4] Dobson S., Kar B., Kumar R., Adams B., Barik S. (2001). A novel tetratricopeptide repeat (TPR) containing PP5 serine/threonine protein phosphatase in the malaria parasite, *Plasmodium falciparum*. BMC Microbiol..

[5] O’Toole N., Hattori M., Andres C., Iida K., Lurin C., Schmitz-Linneweber C., Sugita M., Small I. (2008). On the expansion of the pentatricopeptide repeat gene family in plants. Mol. Biol. Evol..

[6] Tourasse N.J., Choquet Y., Vallon O. (2013). PPR proteins of green algae. RNA Biol..

[7] Chen M.X., McPartlin A.E., Brown L., Chen Y.H., Barker H.M., Cohen P.T. (1994). A novel human protein serine/threonine phosphatase, which possesses four tetratricopeptide repeat motifs and localizes to the nucleus. EMBO J..

[8] Das A.K., Cohen P.W., Barford D. (1998). The structure of the tetratricopeptide repeats of protein phosphatase 5: Implications for TPR-mediated protein-protein interactions. EMBO J..

[9] Blatch G.L., Lassle M. (1999). The tetratricopeptide repeat: A structural motif mediating protein-protein interactions. Bioessays.

[10] Andrea L.D., Regan L. (2003). TPR proteins: The versatile helix. Trends Biochem. Sci..

[11] Sawyer N., Chen J., Regan L. (2013). All repeats are not equal: A module-based approach to guide repeat protein design. J. Mol. Biol..

[12] Barik S. (2019). Protein tetratricopeptide repeat and the companion non-tetratricopeptide repeat helices: Bioinformatic analysis of interhelical interactions. Bioinform. Biol. Insights.

[13] Barik S. (2020). The nature and arrangement of pentatricopeptide domains and the linker sequences between them. Bioinform. Biol. Insights.

[14] Crooks G.E., Hon G., Chandonia J.M., Brenner S.E. (2004). WebLogo: A sequence logo generator. Genome Res..

[15] DeLano W.L. (2002). PyMOL.

[16] Taylor P., Dornan J., Carrello A., Minchin R.F., Ratajczak T., Walkinshaw M.D. (2001). Two structures of cyclophilin 40: Folding and fidelity in the TPR domains. Structure.

[17] Wilson C.G., Kajander T., Regan L. (2005). The crystal structure of NlpI. A prokaryotic tetratricopeptide repeat protein with a globular fold. FEBS J..

[18] Katibah G.E., Lee H.J., Huizar J.P., Vogan J.M., Alber T., Collins K. (2013). tRNA binding, structure, and localization of the human interferon-induced protein IFIT5. Mol. Cell.

[19] Feng F., Yuan L., Wang Y.E., Crowley C., Lv Z., Li J., Liu Y., Cheng G., Zeng S., Liang H. (2013). Crystal structure and nucleotide selectivity of human IFIT5/ISG58. Cell Res..

[20] Szent-Gyorgyi A.G., Cohen C. (1957). Role of proline in polypeptide chain configuration of proteins. Science.

[21] Richardson J.S., Richardson D.C. (1988). Amino acid preferences for specific locations at the ends of alpha helices. Science.

[22] Gunasekaran K., Nagarajaram H.A., Ramakrishnan C., Balaram P. (1998). Stereochemical punctuation marks in protein structures: Glycine and proline containing helix stop signals. J. Mol. Biol..

[23] Lurin C., Andrés C., Aubourg S., Bellaoui M., Bitton F., Bruyère C., Caboche M., Debast C., Gualberto J., Hoffmann B. (2004). Genome-wide analysis of *Arabidopsis* pentatricopeptide repeat proteins reveals their essential role in organelle biogenesis. Plant Cell.

[24] Cheng S., Gutmann B., Zhong X., Ye Y., Fisher M.F., Bai F., Castleden I., Song Y., Song B. (2016). Redefining the structural motifs that determine RNA binding and RNA editing by pentatricopeptide repeat proteins in land plants. Plant J..

[25] Schallenberg-Rüdinger M., Lenz H., Polsakiewicz M., Gott J.M., Knoop V. (2013). A survey of PPR proteins identifies DYW domains like those of land plant RNA editing factors in diverse eukaryotes. RNA Biol..

[26] Shikanai T. (2006). RNA editing in plant organelles: Machinery, physiological function and evolution. Cell. Mol. Life Sci..

[27] Maeda A., Takenaka S., Wang T., Frink B., Shikanai T., Takenaka M. (2022). DYW deaminase domain has a distinct preference for neighboring nucleotides of the target RNA editing sites. Plant J..

[28] Sun Y.K., Gutmann B., Small I. (2019). Non-canonical features of pentatricopeptide repeat protein-facilitated RNA editing in *Arabidopsis* chloroplasts. bioaRxiv.

[29] Schmitz-Linneweber C., Small I. (2008). Pentatricopeptide repeat proteins: A socket set for organelle gene expression. Trends Plant Sci..

[30] Nakamura T., Yagi Y., Kobayashi K. (2012). Mechanistic insight into pentatricopeptide repeat proteins as sequence-specific RNA-binding proteins for organellar RNAs in plants. Plant Cell Physiol..

[31] Knoop V. (2011). When you can’t trust the DNA: RNA editing changes transcript sequences. Cell. Mol. Life Sci..

[32] Fujii S., Small I. (2011). The evolution of RNA editing and pentatricopeptide repeat genes. New Phytol..

[33] Takenaka M., Zehrmann A., Verbitskiy D., Härtel B., Brennicke A. (2013). RNA editing in plants and its evolution. Annu. Rev. Genet..

[34] Yagi Y., Tachikawa M., Noguchi H., Satoh S., Obokata J., Nakamura T. (2013). Pentatricopeptide repeat proteins involved in plant organellar RNA editing. RNA Biol..

[35] Small I.D., Schallenberg-Rudinger M., Takenaka M., Mireau H., Ostersetzer-Biran O. (2020). Plant organellar RNA editing: What 30 years of research has revealed. Plant J..

[36] Ichinose M., Teramoto T., Nakamura I., Shimajiri Y., Yagi Y., Gutmann B. (2025). Fine-tuning of the PPR protein directs the RNA editing activity toward C-to-U or U-to-C conversion. Sci. Rep..

[37] Sugita M., Ichinose M., Ide M., Sugita C. (2013). Architecture of the PPR gene family in the moss *Physcomitrella patens*. RNA Biol..

[38] Gutmann B., Royan S., Schallenberg-Rüdinger M., Lenz M., Castleden I.R., McDowell R., Vacher M.A., Tonti-Filippini J., Bond C.S., Knoop V. (2020). The expansion and diversification of pentatricopeptide repeat RNA editing factors in plants. Mol. Plant.

[39] Macedo-Osorio K.S., Martínez-Antonio A., Badillo-Corona J.A. (2021). Pas de Trois: An overview of penta-, tetra-, and octo-tricopeptide repeat proteins from *Chlamydomonas reinhardtii* and their role in chloroplast gene expression. Front. Plant Sci..

[40] Wang Y., Tan B.-C. (2025). Pentatricopeptide repeat proteins in plants: Cellular functions, action mechanisms, and potential applications. Plant Commun..

[41] Banks J.A., Nishiyama T., Hasebe M., Bowman J.L., Gribskov M., dePamphilis C., Albert V.A., Aono N., Aoyama T., Ambrose B.A. (2011). The *Selaginella* genome identifies genetic changes associated with the evolution of vascular plants. Science.

[42] Rovira A.G., Smith A.G. (2019). PPR proteins—Orchestrators of organelle RNA metabolism. Physiol. Plant..

[43] Barkan A., Small I. (2014). Pentatricopeptide repeat proteins in plants. Annu. Rev. Plant Biol..

[44] Kotera E., Tasaka M., Shikanai T. (2005). A pentatricopeptide repeat protein is essential for RNA editing in chloroplasts. Nature.

[45] Takenaka M., Verbitskiy D., Zehrmann A., Härtel B., Bayer-Császár E., Glass F., Brennicke A. (2014). RNA editing in plant mitochondria—Connecting RNA target sequences and acting proteins. Mitochondrion.

[46] Chun S.O., Garcia E.T., Rejas M., Hayes M.L. (2025). A conserved lysine in an ion-pair with a catalytic glutamate is critical for U-to-C RNA editing but restricts C-to-U RNA editing. Biochemistry.

[47] Salone V., Rüdinger M., Polsakiewicz M., Hoffmann B., Groth-Malonek M., Szurek B., Small I., Knoop V., Lurin C. (2007). A hypothesis on the identification of the editing enzyme in plant organelles. FEBS Lett..

[48] Rüdinger M., Polsakiewicz M., Knoop V. (2008). Organellar RNA editing and plant-specific extensions of pentatricopeptide repeat proteins in jungermanniid but not in marchantiid liverworts. Mol. Biol. Evol..

[49] Ichinose M., Sugita C., Yagi Y., Nakamura T., Sugita M. (2013). Two DYW subclass PPR proteins are involved in RNA editing of ccmFc and atp9 transcripts in the moss *Physcomitrella patens*: First complete set of PPR editing factors in plant mitochondria. Plant Cell Physiol..

[50] Ichinose M., Sugita M. (2018). The DYW Domains of pentatricopeptide repeat RNA editing factors contribute to discriminate target and non-target editing sites. Plant Cell Physiol..

[51] Hashimoto M., Endo T., Peltier G., Tasaka M., Shikanai T. (2003). A nucleus-encoded factor, CRR2, is essential for the expression of chloroplast ndhB in *Arabidopsis*. Plant J..

[52] Nakamura T., Sugita M. (2008). A conserved DYW domain of the pentatricopeptide repeat protein possesses a novel endoribonuclease activity. FEBS Lett..

[53] Yang Y., Ritzenhofen K., Otrzonsek J., Xie J., Schallenberg-Rüdinger M., Knoop V. (2023). Beyond a PPR-RNA recognition code: Many aspects matter for the multi-targeting properties of RNA editing factor PPR56. PLoS Genet..

[54] Takenaka M., Zehrmann A., Brennicke A., Graichen K. (2013). Improved computational target site prediction for pentatricopeptide repeat RNA editing factors. PLoS ONE.

[55] Schmidberger J.W., Bond C.S. (2018). Crystal Structure of a Designer Pentatricopeptide RNA-Binding Protein, Bound to a Complex RNA Target and Featuring an Infinite Superhelix and Microheterogeneity. https://www.wwpdb.org/pdb?id=pdb_00006een.

[56] Yin P., Li Q., Yan C., Liu Y., Liu J., Yu F., Wang Z., Long J., He J., Wang H.-W. (2013). Structural basis for the modular recognition of single-stranded RNA by PPR proteins. Nature.

[57] Yagi Y., Hayashi S., Kobayashi K., Hirayama T., Nakamura T. (2013). Elucidation of the RNA recognition code for pentatricopeptide repeat proteins involved in organelle RNA editing in plants. PLoS ONE.

[58] Ruwe H., Gutmann B., Schmitz-Linneweber C., Small I., Kindgren P. (2019). The E domain of CRR2 participates in sequence-specific recognition of RNA in plastids. New Phytol..

[59] Kobayashi K., Kawabata M., Hisano K., Kazama T., Matsuoka K., Sugita M., Nakamura T. (2012). Identification and characterization of the RNA binding surface of the pentatricopeptide repeat protein. Nucleic Acids Res..

[60] Ban T., Ke J., Chen R., Gu X., Tan M.H., Zhou X.E., Kang Y., Melcher K., Zhu J.K., Xu H.E. (2013). Structure of a PLS-class pentatricopeptide repeat protein provides insights into mechanism of RNA recognition. J. Biol. Chem..

[61] Shen C., Zhang D., Guan Z., Liu Y., Yang Z., Yang Y., Wang X., Wang Q., Zhang Q., Fan S. (2016). Structural basis for specific single-stranded RNA recognition by designer pentatricopeptide repeat proteins. Nat. Commun..

[62] Barkan A., Rojas M., Fujii S., Yap A., Chong Y.S., Bond C.S., Small I. (2012). A combinatorial amino acid code for RNA recognition by pentatricopeptide repeat proteins. PLoS Genet..

[63] Manavski N., Abdel-Salam E., Schwenkert S., Kunz H.H., Brachmann A., Leister D., Meurer J. (2025). Targeted introduction of premature stop codon in plant mitochondrial mRNA by a designer pentatricopeptide repeat protein with C-to-U editing function. Plant J..

[64] Zehrmann A., Verbitskiy D., Härtel B., Brennicke A., Takenaka M. (2010). RNA editing competence of trans-factor MEF1 is modulated by ecotype-specific differences but requires the DYW domain. FEBS Lett..

[65] Zehrmann A., Verbitskiy D., Härtel B., Brennicke A., Takenaka M. (2011). PPR proteins network as site-specific RNA editing factors in plant organelles. RNA Biol..

[66] Verbitskiy D., Zehrmann A., Härtel B., Brennicke A., Takenaka M. (2012). Two related RNA-editing proteins target the same sites in mitochondria of *Arabidopsis thaliana*. J. Biol. Chem..

[67] Kleinknecht L., Wang F., Stübe R., Philippar K., Nickelsen J., Bohne A.V. (2014). RAP, the sole octotricopeptide repeat protein in *Arabidopsis*, is required for chloroplast 16S rRNA maturation. Plant Cell.

[68] Lefebvre-Legendre L., Choquet Y., Kuras R., Loubéry S., Douchi D., Goldschmidt-Clermont M. (2015). A nucleus-encoded chloroplast protein regulated by iron availability governs expression of the photosystem I subunit PsaA in *Chlamydomonas reinhardtii*. Plant Physiol..

[69] Cline S.G., Laughbaum I.A., Hamel P.P. (2017). CCS2, an octatricopeptide-repeat protein, is required for plastid cytochrome c assembly in the green alga *Chlamydomonas reinhardtii*. Front. Plant Sci..

[70] Rahire M., Laroche F., Cerutti L., Rochaix J.-D. (2012). Identification of an OPR protein involved in the translation initiation of the PsaB subunit of photosystem I. Plant J..

[71] Wang F., Johnson X., Cavaiuolo M., Bohne A.V., Nickelsen J., Vallon O. (2015). Two *Chlamydomonas* OPR proteins stabilize chloroplast mRNAs encoding small subunits of photosystem II and cytochrome b6 f. Plant J..

[72] Hillebrand A., Matz J.M., Almendinger M., Müller K., Matuschewski K., Schmitz-Linneweber C. (2018). Identification of clustered organellar short (cos) RNAs and of a conserved family of organellar RNA-binding proteins, the heptatricopeptide repeat proteins, in the malaria parasite. Nucleic Acids Res..

[73] Hollin T., Stajich J.E., Godzik A., Le Roch K.G. (2021). Identification and phylogenetic analysis of RNA binding domain abundant in apicomplexans or RAP proteins. Microb. Genom..

[74] Lee I., Hong W. (2004). RAP—A putative RNA-binding domain. Trends Biochem. Sci..

[75] Chowdhury B., Garai G. (2017). A review on multiple sequence alignment from the perspective of genetic algorithm. Genomics.

[76] Abramson J., Adler J., Dunger J., Evans R., Green T., Pritzel A., Ronneberger O., Willmore L., Ballard A.J., Bambrick J. (2024). Accurate structure prediction of biomolecular interactions with AlphaFold 3. Nature.

[77] Vymětal J., Jakubec D., Galgonek J., Vondrášek J. (2003). Amino Acid Interactions (INTAA) web server v2.0: A single service for computation of energetics and conservation in biomolecular 3D structures. Nucleic Acids Res..

[78] Wang C., Kassem S., Rocha R.E.O., Sun P., Nguyen T.-T., Kloehn J., Liu X., Brusini L., Bonavoglia A., Barua S. (2024). Apicomplexan mitoribosome from highly fragmented rRNAs to a functional machine. Nat. Commun..

[79] Sidik S.M., Huet D., Ganesan S.M., Huynh M.H., Wang T., Nasamu A.S., Thiru P., Saeij J.P.J., Carruthers V.B., Niles J.C. (2016). Genome-wide CRISPR screen in *Toxoplasma* identifies essential Apicomplexan genes. Cell.

[80] Auchincloss A.H., Zerges W., Perron K., Girard-Bascou J., Rochaix J.D. (2002). Characterization of Tbc2, a nucleus-encoded factor specifically required for translation of the chloroplast psbC mRNA in *Chlamydomonas reinhardtii*. J. Cell Biol..

[81] Dauville’e D., Stampacchia O., Girard-Bascou J., Rochaix J.D. (2003). Tab 2 is a novel conserved RNA binding protein required for translation of the chloroplast psaB mRNA. EMBO J..

[82] Barneche F., Winter V., Crevecoeur M., Rochaix J.D. (2006). ATAB 2 is a novel factor in the signalling pathway of light-controlled synthesis of photosystem proteins. EMBO J..

[83] Marx C., Wünsch C., Kück U. (2015). The octatricopeptide repeat protein Raa8 is required for chloroplast trans splicing. Eukaryot. Cell.

[84] Balczun C., Bunse A., Hahn D., Bennoun P., Nickelsen J., Kück U. (2005). Two adjacent nuclear genes are required for functional complementation of a chloroplast trans-splicing mutant from *Chlamydomonas reinhardtii*. Plant J..

[85] Barik S. (2020). The uniqueness of tryptophan in Biology: Properties, metabolism, interactions and localization in proteins. Int. J. Mol. Sci..

[86] Gabler F., Nam S.Z., Till S., Mirdita M., Steinegger M., Söding J., Lupas A.N., Alva V. (2020). Protein sequence analysis using the MPI Bioinformatics Toolkit. Curr. Protoc. Bioinform..

[87] Barik S. (2021). An analytical review of the structural features of pentatricopeptide repeats: Strategic amino acids, repeat arrangements and superhelical architecture. Int. J. Mol. Sci..

[88] Bailey T.L., Johnson J., Grant C.E., Noble W.S. (2015). The MEME Suite. Nucleic Acids Res..

[89] Metz C. (2023). The ‘Godfather of A.I.’ leaves Google and warns of danger ahead. The New York Times.

[90] Jeffrey C. (2020). Enzymes, pseudoenzymes, and moonlighting proteins: Diversity of function in protein superfamilies. FEBS J..

[91] Alvarez-Jarreta J., Amos B., Aurrecoechea C., Bah S., Barba M., Barreto A., Basenko E.Y., Belnap R., Blevins A., Böhme U. (2024). VEuPathDB: The eukaryotic pathogen, vector and host bioinformatics resource center in 2023. Nucleic Acids Res..

[92] Berman H.M., Westbrook J., Feng Z., Gilliland G., Bhat T.N., Weissig H., Shindyalov I.N., Bourne P.E. (2000). The Protein Data Bank. Nucleic Acids Res..

